# *Candida utilis* ATCC 9950 Cell Walls and *β(1,3)/(1,6)*-Glucan Preparations Produced Using Agro-Waste as a Mycotoxins Trap

**DOI:** 10.3390/toxins11040192

**Published:** 2019-03-30

**Authors:** Anna Bzducha-Wróbel, Marcin Bryła, Iwona Gientka, Stanisław Błażejak, Monika Janowicz

**Affiliations:** 1 Faculty of Food Science, Department of Biotechnology, Microbiology and Food Evaluation, Warsaw University of Life Sciences-SGGW, Nowoursynowska Str. 159c, 02-776 Warsaw, Poland; iwona_gientka@sggw.pl (I.G.); stanislaw_blazejak@sggw.pl (S.B.); 2 Prof. Waclaw Dabrowski Institute of Agricultural and Food Biotechnology, Department of Food Analysis, Rakowiecka Str. 36, 02-532 Warsaw, Poland; marcin.bryla@ibprs.pl; 3 Faculty of Food Science, Department of Food Engineering and Process Management, Warsaw University of Life Sciences-SGGW, Nowoursynowska Str. 159c, 02-776 Warsaw, Poland; monika_janowicz@sggw.pl

**Keywords:** *Candida utilis*, cell walls, *β*-glucan, adsorption, waste valorization, aflatoxin B_1_, zearalenone, ochratoxin A, deoxynivalenol, nivalenol, T-2 toxin, fumonisin B_1_

## Abstract

Mycotoxins are harmful contaminants of food and feed worldwide. Feed additives with the abilities to trap mycotoxins are considered substances which regulate toxin transfer from feed to tissue, reducing its absorption in animal digestive tract. Market analysis emphasizes growing interest of feed producers in mycotoxins binders obtained from yeast biomass. The aim of the study was to prescreen cell walls (CW) and *β*(1,3)/(1,6)-glucan (*β*-G) preparations isolated from *Candida utilis* ATCC 9950 cultivated on waste potato juice water with glycerol as adsorbents for aflatoxin B1 (AFB1), zearalenone (ZEN), ochratoxin A (OTA), deoxynivalenol (DON), nivalenol (NIV), T-2 toxin (T-2) and fumonisin B1 (FB1). The adsorption was studied in single concentration tests at pH 3.0 and 6.0 in the presence of 1% of the adsorbent and 500 ng/mL of individual toxin. Evaluated CW and *β*-G preparations had the potential to bind ZEN, OTA and AFB1 rather than DON, NIV, T-2 toxin and FB1. The highest percentage of adsorption (about 83%), adsorption capacity (approx. 41 µg/ g preparation) and distribution coefficient (458.7mL/g) was found for zearalenone when CW preparation was used under acidic conditions. Higher protein content in CW and smaller particles sizes of the formulation could influence more efficient binding of ZEN, OTA, DON and T-2 toxin at appropriate pH compared to purified *β*-G. Obtained results show the possibility to transform the waste potato juice water into valuable *Candida utilis* ATCC 9950 preparation with mycotoxins adsorption properties. Further research is important to improve the binding capacity of studied preparations by increasing the active surface of adsorption.

## 1. Introduction

Mycotoxins are secondary metabolites produced by microscopic fungi, exposure to which causes constant health risk for humans and animals [1]. Agricultural products are mainly contaminated by aflatoxins (AFs), deoxynivalenol (DON), zearalenone (ZEN), ochratoxin A (OTA) fumonisins (FBs) and T-2 toxin, which are generally stable during routine processes applied in food and feed production [1,2,3,4,5].

Due to acute and chronic toxic effects mycotoxins are considered a critical issue in terms of security and safety risks of the feed and food supply chain [1,3,5,6,7]. Numerous efforts are made, including pre-harvest measures, harvest management, and post-harvest strategies, to minimalize the risk of mycotoxins contamination and related economic losses. Even then, their presence in food and animal feed cannot be avoided completely [1,8,9]. Reviews on mycotoxins occurrence in feed materials show that there are still samples that do not comply with the EU requirements for undesirable substances. AFs, DON, ZEN, OTA and FBs are significant in terms of their prevalence in feed and their negative effects on animal performance [1,3,5,8,10,11,12,13].

New, safe and environmentally friendly ways to reduce the exposure of animals to mycotoxins in the feed chain are being developed. One approach is to reduce the toxic effect of mould secondary metabolites on animals using different adsorbents as technological feed additives. They presumably can trap mycotoxins in animals digestive track, reducing their transfer, absorption and systemic toxicity [5,14,15,16,17,18,19].

The possibility of minimization or alleviation of mycotoxin effects on animals by yeast-delivered preparations is an area of present research with promising results [16,20,21,22,23,24]. Yeast-based feed additives may counteract the toxic effect of mould secondary metabolites by acting as adsorbents but also by modulation of local humoral cellular response [24,25,26]. The efficiency of toxins binding by preparations of yeast origin is correlated mostly with the composition, structure and content of polymers which form yeast cell walls [23,27,28,29,30,31]. However, accessible surphase area of the adsorbent is a crucial parameter also [5,32]. *β*-Glucans (*β*(1,3)- and *β*(1,6)-glucan) and mannoproteins are the main structural components of yeast cell wall, while proteins, lipids and chitin are present at lower content [33]. All the mentioned substances have numerous adsorption centers to bind mycotoxins molecules by different adsorption mechanisms including hydrogen bonding, ionic, hydrophobic interactions, electrostatic attraction or coordination bonds [5,20,32,34].

Yeast cell wall structure, the ratio of cell wall polymers and their conformation are a strain-dependent factors. They are also influenced strongly by growth conditions [28,35,36,37]. Until now, the process for mycotoxins binding has been studied mostly for live cells and preparations of *Saccharomyces cerevisiae* origin. Much less is known about complexation of mycotoxins using preparations of other yeast genera.

Our previous studies showed a significant increase in the content of *β*-glucan in cell walls of *Candida utilis* cultivated on agri-food waste - deproteinated potato juice water (DPJW) with the addition of glycerol [36,37]. Yeast cultivation in discussed waste could be a method of its valorization into a preparation with the ability to bind mycotoxins.

The aim of the study was to prescreen at the first time the potential of cell walls and *β*(1,3)/(1,6)-glucan preparations isolated from *Candida utilis* ATCC 9950 biomass cultivated on low cost substrates - waste potato juice water and glycerol, to adsorb different mycotoxins. The single-concentration tests were performed at the range of pH found in the gastrointestinal tract of monogastric animals (pH 3.0 and 6.0).

## 2. Results

### 2.1. Chemical Composition of Studied Cell Walls and β-Glucan Preparations

The water-insoluble cell walls (CW) and purified *β*-glucan (β-G) preparations were isolated from *Candida utilis* ATCC 9950 after biomass cultivation on waste potato juice water and glycerol. The CW preparation was isolated from the biomass by bead milling and consecutive extraction of water-soluble substances, while the purified *β*-G was obtained after CW treatment with isopropyl alcohol, enzymatic protein hydrolysis and autoclaving [36].Tested adsorbents differed in their chemical compositions. The investigated CW preparation had higher protein content (app. 7.6%) compared to the *β*-G preparation (about 2%)—Table 1. Purified *β*-G was composed of app. 93% of total sugar and 85%, *β*(1,3)/(1,6)-glucan, while CW were characterized by a lower content of discussed ingredients. Purified *β*-G preparation consisted mainly of alkali insoluble polysaccharides (75%) and *β*(1,3)-glucan was the main component of discussed fraction (app. 56%)—Table 2.

### 2.2. Microstructure and Particle Size of Isolated Powder Preparations

**Figure 1:**
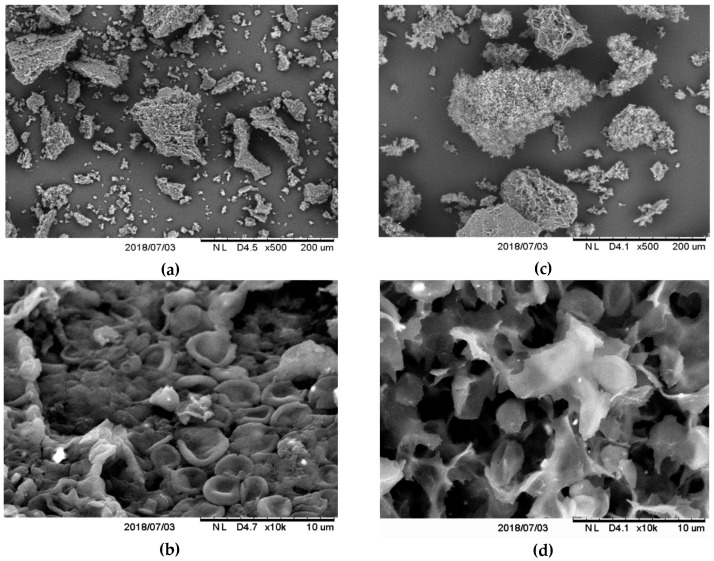
Examples of microscopic structure of studied cell walls (CW) and purified *β*-glucan (*β*-G) particles of *C. utilis* ATCC 9950 obtained by scanning electron microscopy at different magnifications (D—working distance from electron source); (**a**,**b**) morphology of CW particles; (**c**,**d**) morphology of purified *β*-G particles.

**Figure 2:**
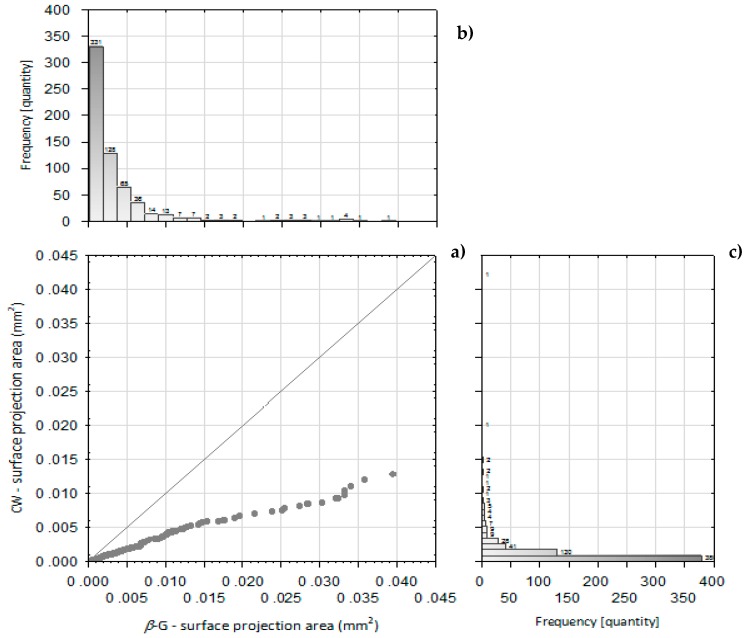
Distribution of the size of the surface projection area of cell walls (CW) and purified *β*-glucan (*β*-G) particles of studied *C. utilis* ATCC 9950. (**a**) comparison of the particles size distribution of CW and *β*-G; (**b**) histogram of particle-size of the surface projected area of purified *β*-glucan (*β*-G); (**c**) histogram of particle-size of the surface projected area of cell walls (CW). The particle size distribution of the particle projection surface for *β*-G and CW debris was prepared on the basis of microscope photographs taken with the Hitachi TM 3000 electron microscope. The plot size distribution charts and histograms were plotted in the STATISTICA 13.3 program by selecting the scatter plot with histograms. Histograms are presented in the coordinate system the frequency of occurrence of specific values in the function of the projection surface area.

The particles microstructure of studied preparations was examined by scanning electron microscopy (SEM). The CW and *β*-G particles obtained significantly differed in morphology (Figure 1a–d). They showed significant agglomeration and formed clumps. Particles of CW consisted of agglomerated micro-residues of yeast cell walls (Figure 1a,b). The morphology of single *β*-glucan particles did not resemble yeast cell wall shape (Figure 1c,d). Computer analysis of SEM images indicated significant differences in particle surface distribution of tested CW and *β*-G preparations (Figure 2), with a wider range found for *β*-G. In order to confirm the results based on the computer image analysis (a subjective assessment of the size of the objects), particle sizes were assessed using the laser diffraction method—Table 3. It was shown that only 10% of CW particles had dimensions up to about 8 μm (similar to the size of yeast cells) while 90% of them belonged to the class with dimensions of up to approx. 200 μm. The studied *β*-G preparation consisted of particles with larger dimensions and D[4,3] diameter compared to CW what indicate more extensive agglomeration. About 10% of *β*-G preparation particles had dimensions of up to 16 μm, 50% up to 85 μm, while 90% of them showed dimensions up to 208 µm. It allowed us to assume that CW preparation had higher active surface area in comparison to purified *β*-G, however both preparations consisted mainly of large particles.

### 2.3. Results of Adsorption Tests

The ability of studied CW and *β*-G preparations of *C. utilis* ATTC 5590 cultivated on agri-waste to adsorb AFB_1_, ZEN, OTA, DON, NIV, FB_1_ and T-2 toxin were summarized in Table 4. The preparations were characterized by the potential to bind ZEN, OTA and AFB_1_ rather than DON, NIV, T-2 toxin and FB_1_. Evaluated *C. utilis* cell walls participated in the higher adsorption of ZEN, OTA, DON and T-2 toxin at proper pH in relation to purified *β*-G preparation. About 82% of ZEN (41 µg/g of preparation) was adsorbed by CW at pH 3.0. Acidic environment favored ZEN binding, regardless of the type of investigated preparation. Cell walls preparation of evaluated *Candida utilis* ATCC 9950 strain was able to bind about 25% of 1000 ng of aflatoxin B_1_ available in tests tubes, at each pH tested. When purified *β*-G preparation was used, the binding level of AFB_1_ was influenced by pH, reaching about 30% at pH 6.0. This corresponded to the adsorption capacity of about 15 µg AFB_1_/g of preparation. Under acidic conditions the process was approx. two times less efficient.

The highest level of OTA binding (approx. 45%) was achieved when CW preparation was applied in adsorption test and the incubation was carried out at pH 3.0. Under mentioned conditions the OTA adsorption capacity reached approx. 22 µg/ g of preparation, while for *β*-G about half less adsorption efficiency was observed. The process of ochratoxin A binding was significantly lower under near-neutral conditions (pH 6.0).

The DON, NIV, FB_1_ and T-2 toxin binding abilities of tested preparations were significantly lower compared to values stated for ZEN, OTA and AFB_1_. The highest percent of DON adsorption (app. 22%) resulted in adsorption capacity about 11 µg DON/g of preparation was noticed after incubation in the presence of cell walls at near-neutral conditions. Less acidic environment favored DON binding to the discussed adsorbent. The highest binding efficiency of T-2 toxin (app. 16%) was stated under the same conditions.

Application of *β*-G preparation allowed to bind approx. 194 ng (19%) of fumoninin B_1_ available in test tubes at pH 3.0. Incubation at pH 6.0 resulted in adsorption at the level of 2% only. Cell walls adsorbed about 12% of FB_1_, regardless of pH. The adsorption efficiency for NIV was only between about 4% at pH 3.0 and about 10% at pH 6.0 (average for CW and *β*-G).

To define the affinity of investigated adsorbing agents for studied mycotoxins the distribution coefficients (Kd) were calculated [38]. The Kd coefficient is defined as the ratio of bound toxin (μg/g) to free toxin (μg/mL) and allows for a quantitative comparison of affinity. The maximum coefficient distribution was stated for CW preparation and ZEN after adsorption at pH 3 (app. 459 mL/ g). When OTA was adsorbed using CW the Kd achieved the level app. 81 mL/ g. The coefficient was app. 41 for *β*-G preparation and AFB_1_ at pH6.The lowest Kd value was stated for *β*-G preparation after FB_1_ binding at pH 6 (app. 2 mL/ g).

## 3. Discussion

The adsorption efficiency of studied preparations was influenced by their chemical characteristics, pH, the type of mycotoxin and presumably by particle size and their structure. The higher protein content in cell walls and much increased surface area of adsorption could be the reason for more efficient complexation of ZEN, OTA, DON and T-2 toxin comparing with purified *β*-glucan. It suggested also that both carbohydrates and protein fractions of cell walls of *C. utilis* were important for adhesion of mentioned toxins, while binding capacity for AFB_1_ was determined by the presence of *β*-glucan polysaccharide. ZEN and OTA are considered nonpolar molecules which are adsorbed by hydrophobic surfaces, while AFB_1_ is more hydrophilic comparing with them [14,39,40,41,42,43]. Cell wall proteins and mannoproteins are associated with the hydrophobicity of yeast cell surface [44]. The adsorption values of AFB_1_ observed under our study were similar to those stated by Pereyra et al. [32] for *S. cerevisiae* cell walls. Higher efficiences of ZEN, OTA and AFB_1_ adsorption at lower concentration of yeast cell walls or *β*-glucan preparations and higher concentrations of toxins in test tubes were presented by other authors [21,40,45,46,47].

It contrasts with our observation, cell walls preparation of *Saccharomyces cerevisiae* origin studied by Tabari et al. [40] were able to bind less OTA comparing with AFB_1_. It may result from different structure of cell wall polysaccharides and different amino acids composition of cell wall proteins present in discussed organelle of *C. utilis* and *S. cerevisiae* strains. The structural characteristic of yeast cell wall polymers depend on yeast strain and growth conditions [30,32,35,36,37,39,48,49]. The possible effect of variability of waste potato juice water and glycerol purity on *Candida utilis* cell wall adsorptive properties deserves to be checked as part of future research therefore.

Considering the requirements for mycotoxin adsorbents [38], the concentration of studied CW and *β*-G preparations was sufficient to bind at least 20% of mycotoxin only in case of ZEN, OTA, AFB_1_ and DON (cell walls). Observed low efficiencies at binding of DON, NIV and FB_1_, which are complex polar structures, are consistent with values found by other authors [50,51,52,53] for different yeast cell wall-delivered preparations. Mycotoxin characteristic determine the affinity to the adsorbent and binding efficiency [21,32,39,40].

The adsorption of mycotoxins by yeast-delivered products may be also influenced by different environmental conditions such as pH, temperature, presence of bile salts and digestive enzymes in gastrointestinal track, feed components, type of ions and their concentration in aqueous solutions, as well as intestinal microbiota activity [15,23,27,32,38,39,42]. Obtained results show lower adsorption of ZEN under near neutral and alkali conditions comparing with acidic conditions regardless of the type of preparations tested (cell walls and *β*-glucan). The same relationship was described in literature [28,50]. Yiannkouris et al. [39] explained that decreased flexibility of *Saccharomyces cerevisiae β*-glucan under acidic pH increased adsorption values of ZEN. In contrast to our results, other authors [50] obtained higher efficiency of ZEN adsorption using *β*-glucan preparation of *Saccharomyces cerevisiae* comparing with cell walls. However, *β*-glucans of different nature did not show the same adsorption properties [32,39,48,49,54].

The physical characteristic of mycotoxin binders, such as the accessible surface area, total charge and its distribution are also important factors influencing the adsorption process [5,20,32]. According to Faucet-Marguis et al. [32] binding efficiency of yeast-based adsorbents should be rather correlated with surface area than with chemical characteristic of the adsorbent. The particle sizes of the preparations isolated in this study were definitely larger comparing with *β*-glucan preparations discussed in literature [55,56]. There is a need to optimize the formulation of studied preparations by reducing the size of the particles. It can presumably improve the binding capacity by increasing their active surface. Agglomerates of yeast cell walls and *β*-glucan particles can be disrupted mechanically or by using ultrasound treatment before drying process [55,56]. At the same time drying methods of yeast cell wall and *β*-glucan isolates also could affect particle microstructure (particle size and shape), interparticle hydrogen bonding and behaviour of the particles in aqueous dispersion [55,57,58]. Lyophylisation used to formulate the preparation studied within the framework of current study could negatively influence their adsorption efficiency. According to literature [57], lyophilisation process causes the distortion of the surface of glucan particles and their compression into sheet layers by strong interactions between particles which resulted in particles aggregation. Presumably, formulation of studied preparations of *Candida utilis* origin by spray-drying would result in improvement in adsorptive properties. The process allow to produce smaller, elliptical and rather compact particles with smooth surface and preserved origin shape and size of the yeast cells with weak agglomeration tendency comparing with freeze-drying [55,58]. Further research aimed at evaluation of the drying process of studied preparations on their mycotoxin binding capacity is important.

Mycotoxin-adsorbent complex have to be stable throughout the entire digestive track [5]. Therefore, gastro-intestinal digestion models in the presence of intestinal microbiota are important to evaluate their influence on studied *C. utilis* cell walls adsorption efficiency and possible desorption of mycotoxins. The in vitro tests using mycotoxin biomarkers would explain whether the possible beneficial effect of *Candida utilis* cell walls preventing mycotoxicosis occurs in the animal body.

## 4. Conclusions

Evaluated cell walls and purified *β*-glucan particles isolated from *Candida utilis* ATCC 9950 cultivated on waste potato juice water showed the ability to bind especially non-polar mycotoxins, like ZEN, OTA and AFB_1_. Studied cell walls showed higher or comparable efficiency of binding of tested mycotoxins in relation to purified *β*-glucan. It encourages the use of non-purified *C. utilis* ATCC 9950 preparations for mycotoxin binding which is economically more beneficial. Further research is important to improve the binding capacity of studied preparation by increasing the active surface of the cell wall formulations. More complex isotherm studies in the presence of feed matrix and optimized cell walls preparation of investigated *C.utilis* strain are the future direction.

## 5. Materials and Methods

### 5.1. Reagents and Analytical Standards

Analytical standards of mycotoxins were purchased from Romer Labs (Tulln, Austria) including: aflatoxin B_1_ (AFB_1_), zearalenone (ZEN), ochratoxin A (OTA), deoxynivalenol (DON), nivalenol (NIV), fumonisin B_1_ (FB_1_) and T-2 toxin (T-2). All standards were dissolved in LC-MS grade acetonitrile to the initial concentration of 100 µg/mL. All mycotoxin solutions were prepared immediately prior to the binding tests.

Other reagents used in this study were: LC-MS-grade metanol (Rathburn Chemicals Ltd., Walkerburn, UK); LC-MS-grade water (Merck, Darmstadt, Germany); LC-MS-grade formic acid (FA) and ammonium formate (Sigma Aldrich, St. Louis, MO, USA); HPLC-grade isopropyl alcohol (Merck, Darmstadt, Germany), analytical grade: glycerol (99.5%), ethanol (96%), NaCl, NaOH, H_2_SO_4_ (95%), Tris-HCl, KCl, Na_2_HPO_4,_ KH_2_PO_4_, glacial acetic acid, sodium lauryl sulfate (Avantor Performance Materials, Gliwice, Poland); 3,5-Dinitrosalicylic acid (Sigma-Aldrich, USA); Pronase E (Sigma-Aldrich, USA); Zymolyase 20T (MP Biomedicals LLC); Yeast Beta-Glucan Kit, K-EBHLG (Megazyme, Ireland); zirconium-glass beads of 1 mm (Biospec Products, USA); high retention cellulose tubing bags for dialysis (Sigma-Aldrich, St. Louis, USA); 0.2-µm mesh, 4-mm-diameter nylon syringe filters purchased from Phenomenex (Torrance, CA, USA).

### 5.2. Production of Cell Walls and β(1,3)/(1,6)-Glucan Preparations

The yeast strain of *Candida utilis* ATCC 9950 was studied as a source of cell walls and *β*(1,3)/(1,6)-glucan preparations. The cultivation medium was composed of waste deproteinated potato juice water (DPJW) with 10% (*w*/*v*) of pure glycerol (99.5%) as a source of carbon with pH 5.0 ± 0.2. Studied preparations were obtained using one lot of waste DPJW for yeast cultivation. The chemical characteristic of the waste and *inoculum* preparation was described in our previous paper [36]. Batch fermentation was performed in 5-L biofermentor (BIOFLO 3000, New Brunswick, USA) with the working volume of 3 L and 10% (*v*/*v*) of inoculum addition. The impeller rotation speed was 300 rev/min, temperature was kept at 28 °C and airflow at 2.5 L/min. Foam was controlled using Acepol 7287 antifoam (Dakis-Biotimex, Poland). The cultivation time was 48 h. Collected biomass was used to isolate cell walls (CW) and *β*(1,3)(1,6)-glucan (*β*-G) preparations of the studied yeast. The detailed procedures of yeast mechanical disintegration using Bead-Beater GB26 homogenisator (Biospec Products, USA) and *β*-glucan extraction were described in our previous work [36]. The preparations of CW and purified *β*-G were tested for total sugar, *β*(1,3)/(1,6)-glucan and protein content. Additionally, the content of alkali soluble and alkali insoluble polysaccharide fractions in purified *β*-G preparation were estimated as well as the content of *β*(1,3)- and *β*(1,6)-glucan insoluble in alkali [36].

### 5.3. Determination of Particle Microstructure and Size of Powdered Cell Walls and β(1,3)/(1,6)-Glucan Preparations

Samples of isolated CW and *β*-G preparations were pre-frozen in an Irinox MOD.51.20 shock cooler (Italy) at an air temperature of −40 °C and stored under freezing conditions for 24 h. The material was then lyophilized in a CHRIST LCG GAMMA 1-16 LSC (United Kingdom) at 63 Pa at the plate temperature of 20 °C. Observation of the morphology and structure of tested CW and *β*-*G* preparations was performed using the Hitachi TM 3000 scanning electron microscope (Japan). Photographs were taken at various magnifications using the MultiScan v. 18.03 program (Computer Scanning Systems II, Poland). Results of the computer image processing allowed to determine the projection surface of the particles. Scatterplots and histograms of the frequency of occurrence of objects with a given projection size per plot were prepared [59].

**Figure 3:**
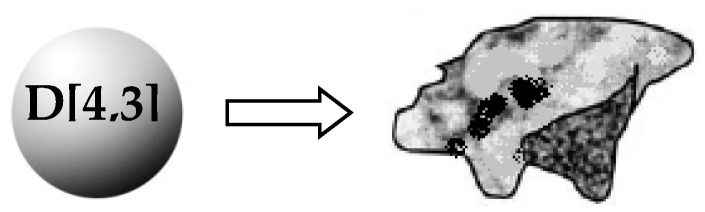
Sphere of diameter D[4,3]: Volume of the sphere which is equal to the average particle volume.

The particle size of the isolated powder preparations was determined by laser diffraction (Cilas 1190 Particle Size, Cilas, France). During the measurement, the powder was dispersed with etanol in the measuring chamber without ultrasound treatment. The particles size was determined at 10, 50 (median) and 90% of volume fraction (the d10, d50 and d90 [μm], respectively). The diameter D[4,3] was defined (diameter of the sphere with the volume equal to the average volume of the particle [μm])—Figure 3.

### 5.4. Conditions of pH-Dependent Mycotoxin Adsorption by Studied Preparations

The PBS buffer used in tests of mycotoxin adsorption was prepared based on NaCl (8 g/L), KCl (0.2g/L), Na_2_HPO_4_ (1.44 g/L), KH_2_PO_4_ (0.24 g/L) dissolved in ultrapure water obtained using Direct-Q 3UV-R water purification system (Merck Millipore, Burlington, Massachusetts, USA). The pH values of PBS buffers were adjusted to 3.0 and 6.0 using HCl solution (app. 18%) (Education Line EL20 pH-meter, Mettler Toledo, Schwerzenbach, Switzeland). Prepared buffers were sterilized for 20 min at 121 °C (HICLAVE HG80 autoclave, HMC Europe) and stored at 4 °C in refrigerator until further use.

The studied preparations of *Candida utilis* cell walls and *β*(1,3)/(1,6)-glucan were suspended in PBS buffer (pH 3.0 and 6.0) for each in vitro test conducted at least in triplicate. The final adsorbent concentration was 10 mg/mL. The efficiency of adsorption of investigated toxins (aflatoxin B_1_, zearalenone, ochratoxin A, deoxynivalenol, nivalenol, fumonisin B_1_ and T-2 toxin) was tested independently in the presence of 500 ng/mL of each mycotoxin in test tubes (1,000 ng in test tube). The applied concentration of tested mycotoxins exceeded significantly the maximum levels of contamination in feed material and feedstuffs by AFB_1_, OTA and T-2 toxin and for complementary and complete feedstuffs in case of ZEN, according to UE directives and guidelines [10,11,12,13].

Two types of negative control samples were prepared: PBS solutions (pH 3.0 and 6.0) with each mycotoxin without the adsorbent and PBS solutions (pH 3.0 and 6.0) with adsorbents but without the mycotoxin. The samples were shaken in a vortex (test tube shaker TTS2, IKA-WERKE Gmbh&Co. Kg) and incubated for 1.5 h at 37 °C in a water bath with shaking (Memmert WNB14, Germany). Following this stage, samples were centrifuged at a room temperature (14,500 rpm/ 5 min, Mini Spin Plus centrifuge, Eppendorf AG, Germany) to separate the binder from the aqueous phase. Next, supernatants were collected in evaporative flasks (5mL volume) and stored at 4 °C before water evaporation was carried out the same day. Samples were evaporated using vacuum evaporation system (Vacuum controler V-855, Vacuum Pump V-700, Heating Bath B-491 and Rotavapor R-215 Büchi, Switzerland) at 40 °C in a water bath at 10 mbar and 210 rpm of rotavapor. The obtained residue was redissolved in a mixture of methanol with water (30:70 ratio) and analysed with LC-MS. Before the analysis, samples were filtered using 0.2-µm mesh nylon syringe filters (Phenomenex, Torrance, CA, USA).

### 5.5. Mycotoxins Analysis with LC-MS

Mycotoxins were analysed with an Acquity H-Class high-performance liquid chromatograph coupled to an LCQ Premiere XE high resolution time-of-flight mass spectrometer (Waters, Milford, MA, USA). Analytes were separated with a UPLC C18 Cortecs chromatographic column (2.1 × 100 mm, 1.6 µm; Waters, Milford, MA, USA) with a pre-column at its front. The mobile phases were composed of methanol:water (90:10, *v*/*v*) (phase A) and methanol:water (10:90, *v*/*v*) (phase B) according to the modified procedure of Bryła et al. [60]. Both phases contained 0.2% formic acid and 5 mM ammonium formate. The flow rate was 0.3 mL/min. The following gradient was used: 1%–50% A from 0 to 8 min; 50%–95% A from 8 to 11 min; constant 95% A from 11 to 22 min; 95%–1% A from 22 to 22.6 min; and constant 1% A from 22.6 to 27 min. Samples of 5 μL were injected into the column. The mass spectrometer was operated in the positive and negative electrospray ionization mode (ESI). The ion source temperature was 150 °C while desolvation temperature was set at 350 °C. The nebulizing gas (nitrogen) flow rate was 750 L/min and the cone gas flow rate was 20 L/min. Capillary bias: 3000 V/2800 V in positive and negative ion modes, respectively. The V mode of the ion optics was used. The mass spectrometer was calibrated using a leu-enkephalin. The following precursor ions (m/z) were registered for individual analytes: AFB_1_—313.1 (M + H)^+^, OTA—404.1 (M + H)^+^, ZEN—317.1 (M − H)^−^, DON—341.2 (M + FA − H)^−^, NIV—357.2 (M + FA − H)^−^, FB_1_—722.4 (M + H)^+^, T-2 toxin—489.2+484.2 (respectively (M + Na)^+^ and (M + NH_4_)^+^.

The relative standard deviations (RSD) were determined and presented in Table 5 for each mycotoxin at concentrations used in the analysis. For mycotoxins that were adsorbed with the efficiency higher than 20% the RSD were determined for two concentrations (the lower concentration was app. below the level of free toxin in solution stated after adsorption).

The analytical technique (UPLC-HRMS) applied for determination of studied mycotoxins is characterized by a narrow dynamic range (a narrow range of concentrations at which the detector response is linear) therefore it was necessary to dilute samples before analysis. Concentrations of working solutions obtained after dilution were in the middle or upper range of the linearity of the method. With respect to limit of quantification, these values were higher by at least 2 orders of magnitude.

### 5.6. Estimation of Adsorption Efficiency, Adsorption Capacity and Distribution Coefficient

The amount of each adsorbed toxin was calculated by substracting the amount of free toxin found in samples after incubation in the presence of adsorbents from the amount of toxin found in the control samples without studied preparations (binder-free sample subjected to all steps of adsorption study). Preliminary studies confirmed that the quantities of each toxin in binder-free samples were the same as in standards solutions of tested mycotoxins (without any pretreatment). It means that there was no degradation of mycotoxins during incubation in PBS buffer, no adsorption to used centrifuge tubes and no mycotoxins losses during sample preparation before analysis.

The adsorption efficiency **(% _ads_)** was calculated using the formula:

(1) $$\%ads=\left(\frac{Co-Caq}{Co}\right)\times 100\;\left[\%\right]$$

where: Co—concentration of mycotoxin in the supernatant of binder-free control (with no adsorbent but subjected to incubation and all procedures of sample preparation before analysis); Caq—residual concentration of mycotoxin in the solution after adsorption test.

Adsorption capacity (*C_ads_*) was calculated as µg of each toxin adsorbed by 1g of dry weight of studied preparation [µg/ g] under tested pH conditions.

Distribution coefficients (*K_d_*) were determined using the formula proposed for single-concentration studies in the scientific report submitted to European Food Safety Authority on mycotoxin-detoxifying agents [38]:

*Kd* = (%_ads_ /%_aq_)/ C_binder_ [mL/g] (2)

where: %_ads_—percentage of the molecule bound to the adsorbing agent (adsorption efficiency); %_aq_—percentage of the molecule remaining free in solution; C_binder_—concentration of adsorbing agent [g/mL].

### 5.7. Data Analysis

Statistical data was evaluated using the STATISTICA V.13.1 software kit (StatPoint Technologies, Inc., Warrenton, VA, USA). An analysis of variance with the one-way ANOVA method (Tuckey’s test) was carried out at the α = 0.05 level of significance to assess the significance of the differences. Statistically homologous groups were marked in tables with the same letter.

## Figures and Tables

**Table 1:** Chemical characterization of cell walls (CW) and *β*(1,3)/(1,6)-glucan preparations (*β*-G) of *Candida utilis* ATCC 9950 studied for mycotoxins adsorption [36].

Preparation	Total Sugars	*β*(1,3)/(1,6)-Glucan	Protein
	[g 100g Preparation]		
CW	68.7 ± 4.0 a^1^	62.9 ± 0.7 a	7.6 ± 0.5 b
*β*-G	93.4 ± 3.1 b	84.8 ± 1.3 b	2.0 ± 1.3 a

^1^ Mean values ± SD; a, b, c… – mean values in column marked with the same letters do not differ significantly, Tukey’s test, α = 0.05.

**Table 2:** The content of alkali soluble and alkali insoluble polysaccharides in *β*-glucan preparation studied for mycotoxins adsorption [36].

Preparation	Alkali Insoluble Polysaccharides	*β*(1,3)-Glucan Insoluble in Alkali	*β(1,6)*-Glucan Insoluble in Alkali	Alkali Soluble Polysaccharides
	[g 100g preparation]			
*β*-G	75.6 ± 2.5	56.2 ± 5.9 (74.3%)*	19.5 ± 0.5 (25.6)*	24.4 ± 2.9

Mean values ± SD; *β*-G: β(1,3)/(1,6)-glucan preparation; *percentage in alkali insoluble polysaccharides.

**Table 3:** Dimensions of lyophilizated cell walls (CW) and *β*-glucan (*β*-G) particles isolated from *C. utilis* ATCC9950 biomass cultivated on agro-waste.

Preparation	d10^*^ [um]	d50^**^ [um]	d90^***^ [um]	D[4,3] ^****^ [um]
CW	7.76 ± 0.56 ^a^	62.58 ± 4.14 ^A^	201.91 ± 15.01 *^A^*	85.42 ± 6.29 *^a^*
*β*-G	16.10 ± 0.46 ^b^	84.98 ± 2.38 ^B^	208.98 ± 4.70 *^B^*	101.52 ± 2.46 *^b^*

Mean values ± SD; *a, b, A, B …—mean values in column marked with the same letters do not differ significantly, Tukey’s test, α = 0.05, p = 0.0002; ^*^d(0.1)—10 % of the particles has dimensions up to the stated value [um]; ^**^d(0.5) – 50 % of the particles has dimensions up to the stated value [um]; ^***^d(0.9)—90 % of the particles has dimensions up to the stated value [um]; ^****^D[4,3]—diameter of the sphere whose volume is equal to an average particle volume [um].

**Table 4:** Results of adsorption of studied mycotoxins by cell walls (CW) and *β*(1,3)/(1,6)-glucan (*β*-G) preparations of *C. utilis* ATCC 9950 in relation to pH of incubation.

Adsorption Characteristic	pH	CW							*β*-G						
		AFB_1_	ZEN	OTA	DON	NIV	T-2	FB_1_	AFB_1_	ZEN	OTA	DON	NIV	T-2	FB_1_
*M_ads_* (ng)	**3.0**	248 ^de^	821 ^h^	446 ^f^	139 ^abcd^	41 ^ab^	72 ^abc^	101 ^abc^	170 ^cde^	743 ^h^	247 ^de^	112 ^abc^	36 ^ab^	106 ^abc^	194 ^cde^
*%_ads_*		25 ^DE^	82 ^H^	45 ^F^	14 ^ABCD^	4 ^AB^	7 ^ABC^	12 ^ABC^	17 ^CDE^	74 ^H^	24 ^DE^	11 ^ABC^	4 ^AB^	11 ^ABC^	19 ^CDE^
*C_ads_* (µg/g)		12 ^lm^	41.1 ^p^	22 ^n^	7 ^ijkl^	2 ^ij^	5 ^ijk^	5 ^ijk^	9 ^klm^	37 ^p^	12 ^lm^	6 ^ijk^	2 ^ij^	5 ^ijk^	10 ^klm^
*K_d_* (mL/g)		33	459	81	16	4	8	13	21	289	32	13	4	12	24
*M_ads_* (ng)	**6.0**	252 ^de^	756 ^h^	117 ^abc^	215 ^cd^	95 ^b^	161 ^bcd^	114 ^abc^	291 ^e^	614 ^g^	174 ^cde^	157 ^bcd^	121 ^abc^	77 ^abc^	21 ^a^
*%_ads_*		25 ^DE^	76 ^H^	12 ^ABC^	22 ^CD^	10 ^B^	16 ^BCD^	13 ^ABC^	29 ^E^	61 ^G^	17 ^CDE^	16 ^BCD^	12 ^ABC^	8 ^ABC^	2 ^A^
*C_ads_* (µg/g)		13 ^lm^	38 ^p^	6 ^ijk^	11 ^kl^	5 ^j^	8 ^jki^	6 ^ijk^	15 ^m^	31 ^o^	9 ^klm^	8 ^jkl^	6 ^ijk^	4 ^ijk^	1 ^i^
*K_d_* (mL/g)		34	310	13	22	11	19	14	41	159	21	19	13	8	2

*M_ads_*—the amount of toxin bound; ***%_ads_***—the percentage of the molecule that is bound to the adsorbing agent (adsorption efficiency, %); *C_ads_*—adsorption capacity (concentration adsorbed by 1 g of preparation); *K_d_*—distribution coefficient. CW cell wall preparation; *β*-G—*β*-glucan preparation. The initial content of mycotoxin was 1000 ng in each test tube. Values represent mean of at least three independent experiments; a, b, c…—mean values marked with the same letters do not differ significantly (Tukey’s test, α = 0.05).

**Table 5:** Relative standard deviations at analyzed concentrations of studied mycotoxins.

Mycotoxin	RSD (%)
AFB_1_	5.1 (*n* = 5; 450 ng/mL) 6.9 (*n* = 5; 250 ng/mL)
ZEN	4.2 (*n* = 5; 450 ng/mL) 12.2 (*n* = 5; 100 ng/mL)
OTA	4.0 (*n* = 5; 450 ng/mL) 7.4 (*n* = 5; 200 ng/mL)
DON	5.5 (*n* = 5; 450 ng/mL)
NIV	4.5 (*n* = 5; 450 ng/mL)
T-2 toxin	4.8 (*n* = 5; 450 ng/mL)
FB1	5.9 (*n* = 5; 450 ng/mL)
